# Fouling and Mitigation Behavior of Foulants on Ion Exchange Membranes with Surface Property in Reverse Electrodialysis

**DOI:** 10.3390/membranes13010106

**Published:** 2023-01-13

**Authors:** Mahamuda Akter, Jin-Soo Park

**Affiliations:** 1 Department of Civil, Environmental and Biomedical Engineering, The Graduate School, Sangmyung University, 31 Sangmyungdae-gil, Dongnam-gu, Cheonan 31066, Republic of Korea; 2 Future Environment and Energy Research Institute, Sangmyung University, 31 Sangmyungdae-gil, Dongnam-gu, Cheonan 31066, Republic of Korea; 3 Department of Green Chemical Engineering, College of Engineering, Sangmyung University, 31 Sangmyungdae-gil, Dongnam-gu, Cheonan 31066, Republic of Korea

**Keywords:** membrane fouling, ion exchange membrane, surface property, foulant, fouling mitigation, reverse electrodialysis

## Abstract

In this study, two different types of ion exchange membranes are used to investigate the tendency of membrane fouling with respect to surface roughness and hydrophilicity. Commercially available membranes reinforced by electrospun nanofiber have rough and hydrophilic surfaces, and lab-made pore-filling membranes exhibit a smooth and hydrophobic surface. Three different organic surfactants (i.e., cationic, anionic and non-ionic surfactants) are chosen as foulants with similar molecular weights. It is confirmed that membrane fouling by electrical attraction mainly occurs, in which anionic and cationic foulants influence anion and cation exchange membranes, respectively. Thus, less fouling is obtained on both membranes for the non-charged foulant. The membranes with a rough surface show a higher fouling tendency than those with a smooth surface in the short-term continuous fouling tests. However, during the cyclic operations of fouling and mitigation of the commercially available membranes, the irregularities of a rough membrane surface cause a rapid increase in electrical resistance from the beginning of fouling due to excessive adsorption on the surface, but the fouling is easily mitigated due to the hydrophilic surface. On the other hand, the membranes with a smooth surface show alleviated fouling from the beginning of fouling, but the irreversible fouling occurs as foulants accumulate on the hydrophobic surface which causes membrane fouling to be favorable.

## 1. Introduction

In recent years, reverse electrodialysis (RED) has become a very attractive technology to capture salinity gradient energy from mixing seawater with fresh water (i.e., generating direct electricity from salinity gradients and the lowest fouling properties) [1,2]. Fouling is one of the most common problems in electrodialysis (ED) using ion exchange membranes (IEMs). In general, fouling is caused by the precipitation of foulants such as organics, colloids and biomass into IEMs and/or onto the surface of IEMs [3,4,5,6,7,8,9,10,11,12,13,14]. The fouling causes a decrease in the transport flux of ions due to fouling complications of the membrane, an increase in the membrane resistance and a loss in selectivity and thus affects negatively membrane properties and performance [15,16]. There are several types of fouling, for example, organic fouling (e.g., carbohydrates, proteins or oil), colloidal fouling (e.g., silica, aluminum oxide and iron oxide), biofouling (e.g., plants, algae and small animals) and scaling fouling (i.e., inorganic salts) in water circuits. All the fouling behaviors are subjected to physical (hydrodynamic), chemical and/or electrochemical interconnection between IEMs and foulants. The aforementioned fouling occurs when the foulants that exist in the feed solution pass through the membrane surface. The foulants affect not only the membrane surface but also the flow channels of the processes using IEMs since scaling and colloidal fouling block the feed water channel, resulting in an increase in the pressure drop in the feed compartment and a reduction in the flow distribution. The stack configuration of ED and RED processes are essentially the same. The RED stacks consist of anion exchange membranes (AEM) and cation exchange membranes (CEMs), low and high salinity water compartments and anode and cathode electrodes. Thus, membrane fouling issues raised in conventional ED also need to be addressed in RED.

Both AEMs and CEMs are affected by several types of foulants. It is mainly related to the electrostatic interaction between charged foulants and IEMs with oppositely charged surfaces. Since AEMs and CEMs have negatively and positively charged functional groups, respectively, oppositely charged foulants could be electrostatically attracted to their surfaces [17]. In a recent study, several types of organic foulants, i.e., charged and non-charged species, were investigated to confirm three different fouling pathways, i.e., electrostatic attraction, electromigration and the macromolecule interaction between IEMs and foulants in a RED system [18,19]. Foulants exist in rivers and seawater with low and high salt concentrations, respectively, in RED. Hence, previous studies have reported the effect of foulants in rivers and/or seawater on the electrical resistance of AEMs and CEMs in RED. The different fouling behaviors of IEMs have been observed depending on the type of streams in which foulants were present. As a result, the worst fouling behavior was observed when the charged foulants were present in river streams. It was because the zeta potential of charged foulants in bulk solutions decreased due to the higher ionic strength of seawater streams, resulting in a lowering of the net electrostatic effect [18]. Y. Oh et al. reported the effect of divalent cations (e.g., magnesium, calcium and barium) on the RED performance in terms of open-circuit voltage and maximum power density. As a result, it was found that divalent cations with a smaller hydrated radius showed higher electrical resistance in the static mode and the increased electrical resistance of CEMs resulted in power reduction during the continuous operation of the bench-scale RED process [20]. It reveals that physical–chemical interactions are also dependent on the interaction between IEMs and foulant properties. In other words, it means that physicochemical properties of foulants such as the concentration, particle size and zeta potential of foulants in the feed solution also act as an unfavorable character in IEMs. Another recent study has reported that the roughness of the membrane surface influenced the fouling tendency. Fouling behavior was investigated in RED using roughness-controlled IEMs with natural seawater and river water. They proposed spacerless stacks using profiled membranes to mitigate fouling tendency [21]. According to the aforementioned fouling studies of IEMs in RED, it is reasoned that the fouling of IEMs significantly depends on the membrane surface characteristics such as hydrophobic/hydrophilic, roughness, and electrostatic charge properties and the physicochemical properties of foulants. A deep insight into the behavior of membrane fouling could facilitate the development of the technology of membrane fouling mitigation. Up to now, several mitigation techniques to mitigate the aforementioned IEMs’ fouling were introduced such as physical/mechanical, electrochemical and chemical cleaning methods [14,18,22,23,24]. However, not all the methods are effective in the mitigation of IEMs’ fouling. The optimal pairing between the fouling behavior and the mitigation method should be determined or developed.

In this study, two different types of IEMs are used to investigate the fouling tendency to different surface roughness and hydrophilicity of the IEMs. Commercially available IEMs are reinforced by electrospun nanofiber having rough and hydrophilic surfaces and lab-made IEMs are pore-filling membranes having smooth and hydrophobic surfaces. Three different organic surfactants (i.e., cationic, anionic and non-ionic surfactants) with similar molecular weight (MW) are chosen as foulant with the least contributing chemicals to pH and ion conductivity to minimize the change in the chemistry of the membrane surface and bulk solution. Finally, the effect of the surface property of IEMs on fouling and mitigation behavior is investigated. The flowing method of concentrated NaCl solutions is used for fouling mitigation technology as suggested in the previous study [18,19].

## 2. Materials and Methods

**Figure 1:**
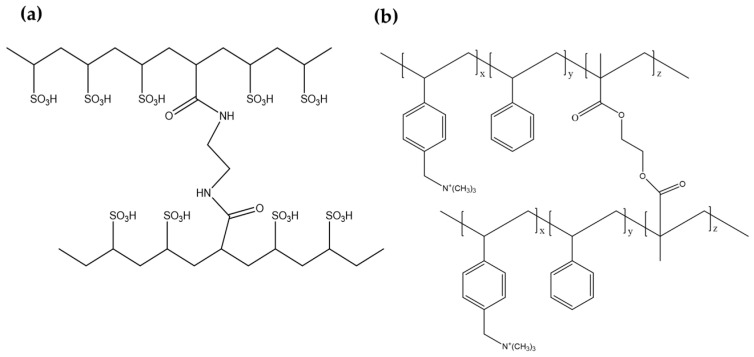
Chemical structures of ion exchangeable polymers of two lab-made ion exchange membranes: (**a**) crosslinked poly (ethylenesulfonic acid) and (**b**) quaternized poly (styrene-co-ethylene glycol dimethacrylate).

Two commercially available and two lab-made pore-filling AEMs and CEMs were used in two different sets of a bench-scale RED stack. Fujifilm^®^ AEM (Type 1) and CEM (Type 10) were purchased and used as received. Their main properties are summarized in Table 1. The lab-made pore-filling AEM and CEM were prepared as described in our earlier studies, respectively [25,26]. The chemical structures of two lab-made AEM and CEM are illustrated in Figure 1. The AEM and CEM are based on quaternized poly (styrene-co-ethylene glycol dimethacrylate) and crosslinked poly (ethylenesulfonic acid), respectively. The properties of the lab-made IEMs used in this study are summarized in Table 1. The thickness of the dry IEMs was measured by a digital thickness gauge (547-520S, Mitutoyo, Schenectady, NY, USA). For the areal membrane resistance (AMR) of the IEMs equilibrated in 0.5 M NaCl for 24 h, the through-plane resistance of the IEMs and the blank solution of 0.5 M NaCl were measured by using a clip cell and a potentiostat/gavanostat with a frequency response analyzer (SP-150, Bio-Logic Science Instruments, Seyssinet-Pariset, France), which is scanned from 1 MHz to 1 mHz with amplitude of 10 mV and was calculated using the equation:

AMR = (|*Z*|*_sample_* · cos*θ_sample_* − |*Z*|*_blank_* · cos*θ_blank_*) × A (1)

where |*Z*|*_sample_* and |*Z*|*_blank_* is the absolute impedance of the membrane samples and the blank solution at the lowest phase angles, respectively, *θ_sample_* and *θ_blank_* are the phase angles of the membrane samples and the blank solution and *A* is the area of the electrode of a clip cell. The apparent transport number of the IEMs was determined by an electromotive force method in a two-compartment cell mounted by a pair of Ag/AgCl reference electrodes in Haber Luggin capillary and was calculated by the following equation:

(2) $$E_{m}=\frac{\mathrm{RT}}{F}\left(1-2t_{-}\right)ln\frac{C_{1}}{C_{2}}$$

where *E_m_* is the cell potential, R is the universal gas constant (8.3145 J K^–1^ mol^–1^), T is the absolute solution temperature, F is the Faraday constant (96485 C e^–^ mol^–1^) and *C*_1_ and *C*_2_ are the solution concentrations of each compartment. The AMR and transport numbers of all the IEMs were measured five times and averaged. The contact angle on the IEMs’ surface for the evaluation of the hydrophilicity of IEMs was measured by the contact angle measurement system (TL 101, Theta Lite Optical, Biolin Scientific, Gothenburg, Sweden).

Negatively, positively, and non-charged substances with similar MW are used in this study as foulants and are summarized in Table 2. For the selection of cationic and anionic foulants with the least contributing chemicals to pH and ion conductivity, six different foulants (three candidates of each foulant) were examined. Sodium dodecyl sulfate (SDS_288_AS), sodium dodecylbenzenesulfonate (SDBS_348_AS) and sodium dioctyl sulfonsuccinate (SDS_445_AS) for cationic foulants and dodecyltrimethylammonium chloride (LTMAC_264_CS), hexadecltrimethylammonium chloride (CTMAC_320_CS) and cetylpyridinium chloride (CC_340_CS) for anionic foulants were used. In addition, humic acid (53680, Aldrich, USA) was used as a reference for the other foulants used in this study to evaluate the intensity of membrane fouling. The common concentration of a foulant is 0.1 wt.% in a river water stream of 0.017 M NaCl as concentrate. Another stream corresponds to a dialysate (or a seawater stream, 0.513 M NaCl). Thus, all the fouled IEMs used in this study were equilibrated in the solutions containing each foulant of 0.1 wt.% in 0.017 M NaCl for 24 h prior to the measurement of IEMs’ properties or the use in a bench-scale RED stack. The pH and ionic conductivity of all the foulant solutions were measured by a pH and ionic conductivity meter (Orion Star A215, Thermo Fisher Scientific, Waltham, MA, USA). All the IEMs for the measurement of electrical resistance or of RED performance were equilibrated in a corresponding solution for 24 h.

**Figure 2:**
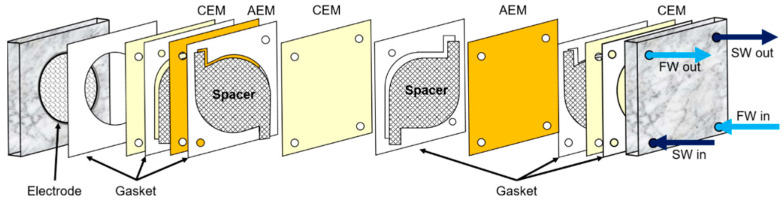
Schematic diagram of a bench-scale RED system. Reprinted with permission from [20]. Copyright 2018 American Chemical Society.

A bench-scale RED stack is composed of twenty cell pairs (twenty-one CEMs and twenty AEMs), polytetrafluoroethylene (PTFE) spacers (0.1 mm thickness) between the membranes, and a pair of platinum-coated titanium mesh electrodes (50 mm diameter, Sung Wing Technology Co., Hong Kong, China) at both ends of a bench-scale RED stack as described in the previous study [20,27]. It was operated at room temperature (22 ± 2 °C). A bench-scale RED stack is illustrated in Figure 2.

Two main streams and one electrode stream were fed to the bench-scale RED stack. Two synthetic feed streams, i.e., seawater with 0.513 M NaCl and river water of 0.017 M NaCl + 0.1 wt.% foulant were fed at 2.5 mL min^–1^. The electrode solution of 0.05 M K_3_[Fe(CN)_6_] (Junsei Chemical Co., Japan) and 0.05 M K_4_[Fe(CN)_6_] (Junsei Chemical Co., Japan) in 1 M Na_2_SO_4_ (Junsei Chemical Co., Japan) was circulated through the bench-scale RED stack at 50 mL min^–1^.

The electrical resistance in the bench-scale RED stack has been measured by connecting a pair of Pt wires inserted at both sides of the target position. The procedure for the measurement of the electrical resistance of a pair of a RED cell comprising of a AEM and a CEM during the combinational operations of fouling and mitigation was as follows: firstly, the RED stack was equilibrated and activated by circulating 0.513 M NaCl as seawater and 0.017 M NaCl as river water for 15 min and then the electrical resistance of the target was measured for 1 min as initial value; secondly, the RED operation was carried out with a 0.513 M NaCl as seawater and a 0.017 M NaCl + 0.1 wt.% foulant solution as river water for 1 h and the electrical resistance of the target was measured when RED operation was stopped; thirdly, the RED operation was resumed and repeated by the second step until the relative error of the performance difference between adjacent two RED operations was reached below 5%; fourthly, the flowing of high concentration of NaCl solutions (i.e., 0.513, 1.026 and 2.565 M NaCl) was performed as fouling mitigation for 1 h and the electrical resistance of the target was measured when mitigation operation was stopped; fifthly, the flowing was resumed and repeated by the fourth step until the relative error of the difference in electrical resistance between adjacent two mitigation operations was reached below 5%; and sixthly, the procedure from the first to the fifth step was repeated.

## 3. Results and Discussion

**Figure 3:**
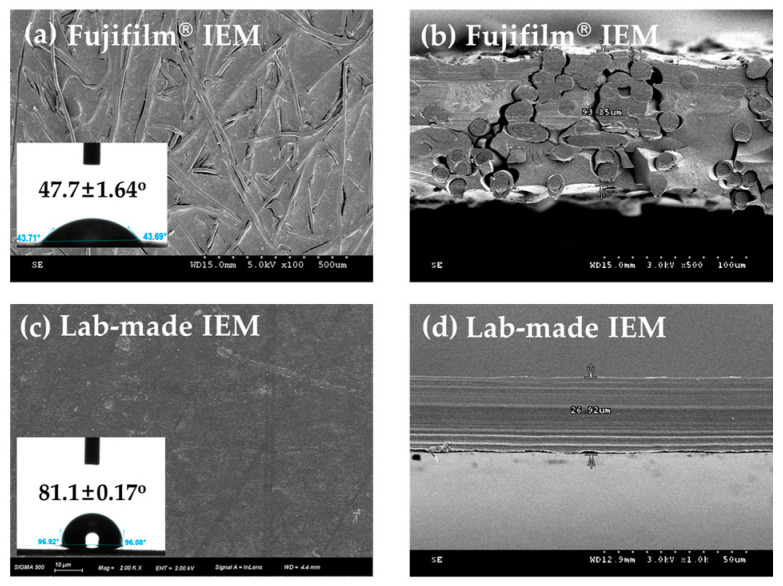
SEM images of (**a**) the top and (**b**) the cross-sectional view of the Fujifilm^®^ IEM and of (**c**) the top and (**d**) the cross-sectional view of the lab-made IEM. The insets in (**a**,**c**) are the contact angles on each IEM.

In this study, the four different IEMs summarized in Table 1 have been investigated to evaluate the effect of the surface properties of the IEMs on fouling and mitigation behavior. Figure 3 shows the scanning electron microscope (SEM) images of the top and cross-sectional view of the four different IEMs. The Fujifilm^®^ IEM shows a rough surface (Figure 3a) and the skeleton of the electrospun polyolefin substrate was exposed randomly in the cross-sectional image (Figure 3b). In contrast, the lab-made IEM shows a very smooth surface and cross-section even though a porous polyolefin substrate was used. It can be attributed to the structural difference of the porous substrates. The Fujifilm^®^ IEMs use three-dimensional structured nanofibers made using the electrospinning method with a diameter ranging from 40 nm to 25 μm to manufacture the membrane [28], whereas the lab-made IEMs use a porous support with a very uniform pore distribution (36.5 ± 2.33 μm) to prepare the membrane. In addition, the contact angle of the IEM surface confirms that the Fujifilm^®^ IEMs are more hydrophilic than the lab-made IEMs. It is due to the difference in membrane preparation methods. Fujifilm^®^ IEMs use a manufacturing method in which ion exchangeable polymer is coated on three-dimensional structured nanofibers used as a skeleton inside the membranes. Thus, the surface of the membranes is completely coated by the ion exchangeable polymer, resulting in a hydrophilic surface. On the other hand, the lab-made IEMs are prepared by filling UV crosslinkable electrolyte and crosslinking agent monomers into the porous substrate, carrying out UV curing, and then polishing the membrane surface to remove brittle polymers unfilled in the substrate, resulting in a hydrophobic surface where a significant amount of the hydrophobic polyolefin support is exposed externally.

**Figure 4:**
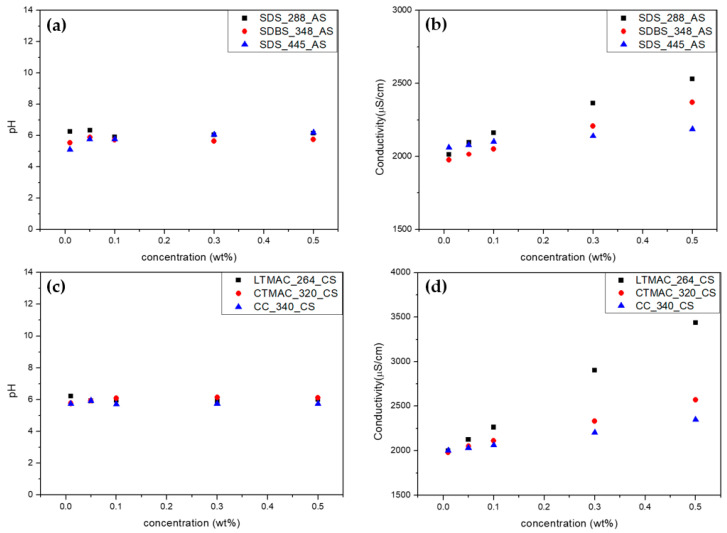
Variation of the pH and ionic conductivity of the solutions containing the foulants with various concentrations in 0.017 M NaCl: (**a**) pH and (**b**) ion conductivity of the anion foulant solutions and (**c**) pH and (**d**) ion conductivity of the cation foulant solutions.

The common foulants release proton or hydroxyl ions or are ionized to increase ionic conductivity. Thus, it should be considered when fouling tests using solutions containing foulants are performed. In order to select the membrane foulants that have the least effect on the pH and ionic conductivity of the solution, the pH and ionic conductivity with the concentration of all the foulants were investigated. Figure 4 shows the variation of the pH and ionic conductivity of the solutions containing anionic and cationic foulants in 0.017 M NaCl with respect to the concentration of foulants. All the foulants show almost constant pH with the concentration of foulants in 0.017 M NaCl. It is confirmed that there is no proton or hydroxyl ion source for all the foulants. In other words, all the anionic and cationic foulants are well equilibrated by sodium and chloride, respectively. However, the ionic conductivity of the foulant solutions linearly increases as the concentration of the foulants increases. It is because the counter ions, i.e., sodium and chloride ions for the anionic and cationic foulants, respectively, are fully dissociated in a 0.017 M NaCl solution to contribute an increase in ionic conductivity. Up to the foulant concentration of 0.1 wt.%, the foulants with the least increment in ionic conductivity could be the most appropriate for the anionic and cationic foulants that have less impact on the water chemistry of the streams of RED. Thus, SDBS_348_AS and CC_340_CS have been finally chosen.

**Figure 5:**
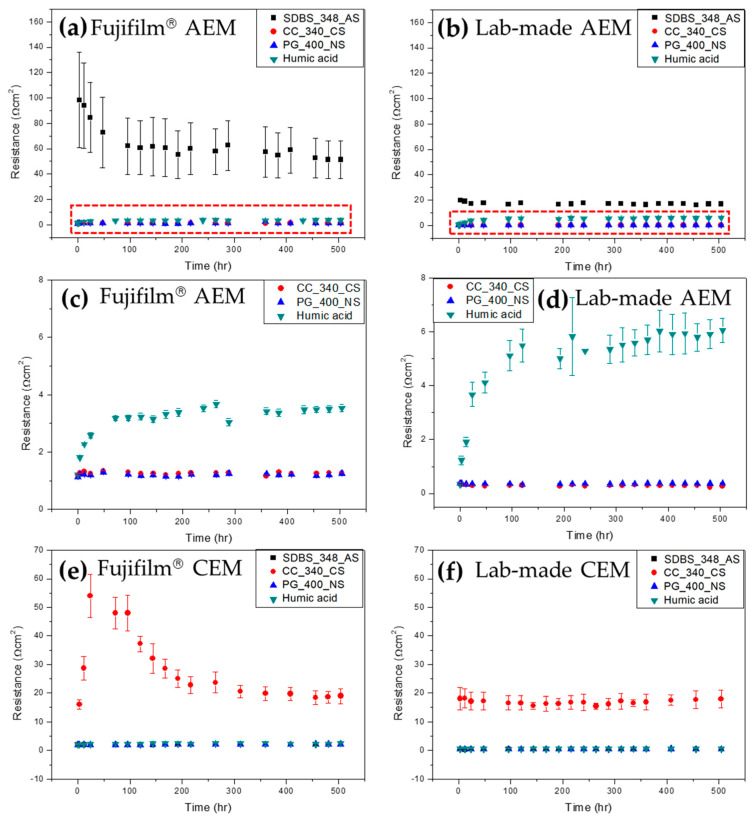
Variation of the electrical resistance of the four different IEMs in the different foulant solutions (SDBS_348_AS, CC_340_CS, PG_400_NS and humic acid): (**a**) Fujifilm^®^ AEM, (**b**) lab-made AEM, (**c**) the enlarged graph of the red dotted square in (**a**), (**d**) the enlarged graph of the red dotted square in (**b**), (**e**) Fujifilm^®^ CEM and (**f**) lab-made CEM.

**Figure 6:**
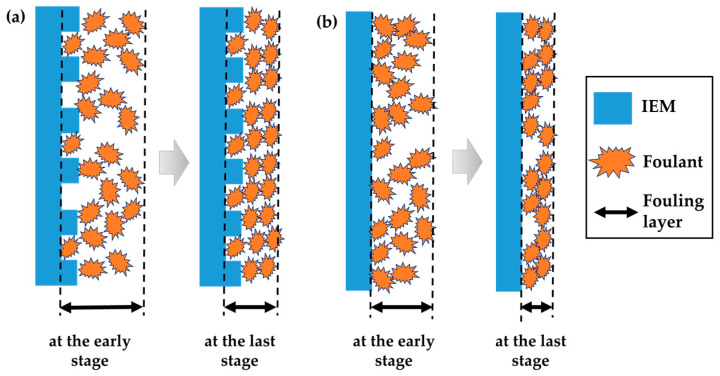
Illustration of describing the change in the fouling behavior on (**a**) the Fujifilm^®^ IEMs with the rough and hydrophilic surface and (**b**) the lab-made IEMs with the smooth and hydrophilic surface at the early (left-hand side) and the last stage (right-hand side) for 500 h immersing in the SDBS_348_AS + 0.017 M NaCl.

Figure 5 shows the variation of electrical resistance of the four different IEMs immersed in 0.1 wt.% foulant + 0.017 M NaCl solutions. The foulants were introduced only in river water (0.017 M NaCl) to foul the IEMs effectively since the previous studies have reported the mitigation behavior of IEMs’ fouling in a NaCl solution of high concentration such as seawater (0.513 M NaCl) [18,19]. As shown in Figure 5a,b,e,f, the anionic foulant, SDBS_348_AS, causes only the AEMs to increase electrical resistance and the cationic foulant, CC_340_CS, results in an increase in electrical resistance only for the CEMs. The non-charged foulant, PG_400_NS, contributes less fouling to both the AEMs and the CEMs. Figure 5c,d show the enlarged variation in electrical resistance of the membranes in the solutions containing the positive, non-charged foulant and humic acid. Known as a typical anionic membrane foulant, humic acid causes an increase in the electrical resistance of the AEMs. It is noted that the increment in electrical resistance of the AEMs by SDBS_348_AS shows higher than that by humic acid. It implies that SDBS_348_AS could be used as a good anionic fouling substance for AEMs. As shown in Figure 3, the Fujifilm^®^ IEMs are much rougher and hydrophilic than the lab-made IEMs. Figure 5a and b show significantly different fouling behavior for the AEMs in the 0.1 wt.% SDBS_348_AS + 0.017 M NaCl solution. More aggravated fouling was obtained for the Fujifilm^®^ AEM (36–137 Ω⋅cm^2^) than the lab-made AEM (~18 Ω⋅cm^2^). Moreover, it reveals that the higher deviation in electrical resistance for the Fujifilm^®^ AEM represents a dynamic process between bound and unbound SDBS_348_AS possibly involving the rougher and hydrophilic surface of the Fujifilm^®^ AEM yet to reach a steady state or equilibrium up to 500 h, while the fouling of the lab-made AEM shows almost constant electrical resistance from the beginning due to the smooth and hydrophobic membrane surface as shown in Figure 5b. This fouling behavior is almost the same in the CEMs as shown in Figure 5e,f. However, it is observed that the equilibrium is reached at 300 h and a similar electrical resistance is finally obtained for the CC_340_CS fouling on the Fujifilm^®^ CEM, compared to the lab-made CEM. The variation of electrical resistance showing the uphill at the early stage of fouling up to ~100 h for the Fujifilm^®^ due to rapid adsorption between irregularities on the rough membrane surface and the following saturation at the last stage after 200 h is observed for the Fujifilm^®^ and the lab-made IEMs in Figure 5e,f. This possible fouling mechanism is illustrated in Figure 6 for a better understanding of the aforementioned discussion for Figure 3 and Figure 5. For the Fujifilm^®^ IEMs, loose and thick fouling layers could be formed due to the adsorption between irregularities formed on the rough surface at the early stage of fouling and dense fouling layer might be formed due to accumulative deposition as illustrated in Figure 6a. The thickness of the fouling layer would be thicker than the lab-made IEMs due to the irregularities. The thicker thickness might cause higher electrical resistance. For the lab-made IEMs, loose and thin fouling layers could be formed due to the smooth surface and a dense fouling layer might be also formed due to accumulative deposition as illustrated in Figure 6b. Many studies reported that hydrophilicity can induce anti-fouling properties, as it provides an energy barrier that makes the adsorption of ionic foulants unfavorable [29,30,31,32,33]. However, the fouling impact on the irregularities was greater than that on the hydrophilicity in this study since the fouling of the Fujifilm^®^ IEMs with rough and hydrophilic surfaces was more severe than the lab-made IEMs with smooth and hydrophobic surfaces under the continuous fouling environment.

**Figure 7:**
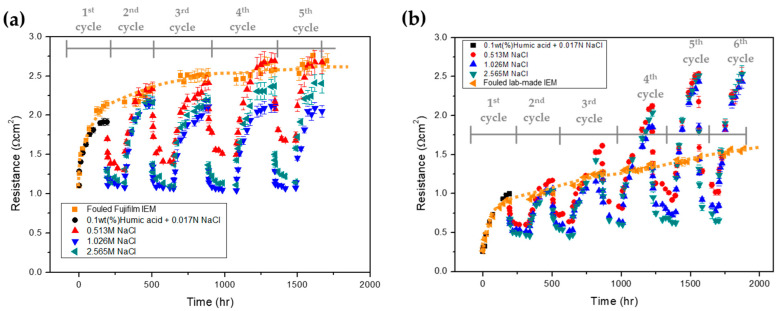
Variation of the averaged electrical resistance of a cell pair using (**a**) the fouled Fujifilm^®^ and (**b**) the fouled lab-made IEMs in a bench-scale RED stack (the dotted lines represent the variation of electrical resistance for the fouling in no presence of mitigation).

Finally, the fouling mitigation of the IEMs with different surface properties used in this study was investigated. The previous studies reported that the high concentration of a NaCl solution such as seawater effectively mitigated the membrane fouling formed by electrostatic attraction between ionic foulants and IEMs [18,19]. Thus, the flowing of a concentrated NaCl solution into a dialysate and a concentrate between RED operations was used as a fouling mitigation method in this study. Figure 7 shows the variation of the average electrical resistance calculated by dividing the electrical resistance of a cell pair of RED (one AEM and one CEM) by the number of IEMs in a cell pair during the cyclic operation of fouling and mitigation in the bench-scale RED stack. A total of five and six cyclic operations were performed for the fouled Fujifilm^®^ IEMs and the fouled lab-made IEMs, respectively. The dotted lines represent the variation of electrical resistance of a cell pair of RED under continuous fouling in no presence of mitigation operation. Both lines show the saturation behavior, but the rough and hydrophilic Fujifilm^®^ IEMs show a higher increase in electrical resistance. For each cyclic operation in the bench-scale RED stack using the fouled Fujifilm^®^ IEMs, the final electrical resistance of a cell pair of RED was recovered to the initial electrical resistance and did not exceed the electrical resistance of the continuous fouling for the rough and hydrophilic Fujifilm^®^ IEMs as shown in Figure 7a. Among the concentrations of a NaCl solution for flowing as a mitigation technique, 1.026 M NaCl shows the most effectiveness for fouling mitigation. On the other hand, the maximum electrical resistance of each cycle up to the second cycle is maintained under the electrical resistance of the continuous fouling for the fouled lab-made IEMs. As shown in Figure 7b, the higher maximum electrical resistance from the third cycle is obtained than that of the continuous fouling even though the NaCl flowing as fouling mitigation has been performed. As discussed earlier, the Fujifilm^®^ IEMs with the rough surface showed a higher fouling tendency than the lab-made ones with the smooth surface in short-term continuous fouling (i.e., ~500 h). However, a few studies revealed that hydrophilicity hindered membrane fouling [29,30,31,32,33]. In other words, the irregularities on the rough Fujifilm^®^ IEM surface cause severe fouling, but the hydrophilic surface results in reversible fouling. In addition, the lab-made IEM gives rise to minor fouling at the beginning due to the smooth surface, but the irreversible fouling occurs as fouling accumulates due to hydrophobicity which causes membrane fouling favorable.

## 4. Conclusions

In this study, the effect of the surface property, i.e., surface roughness and hydrophilicity, of IEMs on fouling and mitigation behavior was investigated. The four different IEMs were investigated to evaluate the effect of the surface properties of IEMs on fouling and mitigation behavior. The Fujifilm^®^ IEMs show rough and hydrophilic surfaces. In contrast, the lab-made IEMs show very smooth and hydrophobic surfaces. Three different cationic, anionic and non-ionic surfactants were used as foulant. The mitigation method was the flowing method of the concentrated NaCl solutions in a bench-scale RED stack. As a result, it was confirmed that the charged foulants only influenced the IEMs with oppositely charged functional groups. For the IEMs with rough surfaces, loose and thick fouling layers could be formed due to the adsorption between irregularities formed on the rough surface at the early stage of fouling and a dense fouling layer might be formed due to accumulative deposition. For the IEMs with smooth surfaces, loose and thin fouling layers could be formed due to the smooth surface and dense fouling layers might be also formed due to accumulative deposition. In short-term continuous fouling, the rougher surface of the IEMs greatly exhibited worse membrane fouling. However, in the cyclic operation of fouling and mitigation, it was found that the irregularities on the IEM with a rough surface caused more severe fouling, but the hydrophilic property resulted in reversible fouling. Thus, it was easily mitigated. The IEMs with smooth surfaces gave rise to minor fouling at the beginning of the operation, but the irreversible fouling occurred as fouling accumulated due to hydrophobicity which caused membrane fouling to be favorable. Thus, it could be concluded that smooth and hydrophilic IEM surfaces might show an anti-fouling tendency in short- and long-term fouling conditions with cyclic mitigation operation.

## Figures and Tables

**Table 1:** Properties of ion exchange membranes used in reverse electrodialysis [14,18,22].

Ion Exchange Membranes	Fujifilm		Lab-Made	
	AEM (Type 1)	CEM (Type 10)	AEM	CEM
Reinforcement	polyolefin	polyolefin	polyolefin	polyolefin
Thickness (μm)	125	135	28	27
Areal membrane resistance ^1^ (Ω-cm^2^)	1.3 ± 0.04	2.0 ± 0.03	0.30 ± 0.01	0.37 ± 0.008
Transport number ^2^	0.92 ± 0.01	0.99 ± 0.002	0.96 ± 0.008	0.98 ± 0.003

^1^ Measured at 0.5 M NaCl. ^2^ Measured at 0.05–0.5 M NaCl.

**Table 2:** Summary of surfactants as foulants used in this study.

Foulants	Anionic Foulant	Cationic Foulant	Non-Ionic Foulant
Name	sodium dodecylbenzenesulfonate	cetylpyridinium chloride	polyethylene glycol 400
Molecular weight (g mol^−1^)	348.5	340	380–420
Molecular formula	C_18_H_29_NaO_3_S	C_21_H_38_ClN	H(OCH_2_CH_2_)_n_OH
Chemical structure	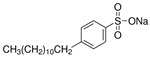	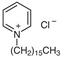	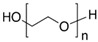

## Data Availability

Data sharing not applicable.
